# Chemoresistance in the Human Triple-Negative Breast Cancer Cell Line MDA-MB-231 Induced by Doxorubicin Gradient Is Associated with Epigenetic Alterations in Histone Deacetylase

**DOI:** 10.1155/2019/1345026

**Published:** 2019-06-02

**Authors:** Jeonghun Han, Wanyoung Lim, Daeun You, Yisun Jeong, Sangmin Kim, Jeong Eon Lee, Tae Hwan Shin, Gwang Lee, Sungsu Park

**Affiliations:** ^1^Regenerative Medicine and Cell Therapy Institute, Seoul National University Bundang Hospital, Seongnam 13620, Republic of Korea; ^2^School of Mechanical Engineering, Sungkyunkwan University, Suwon 16419, Republic of Korea; ^3^Department of Biomedical Engineering, Sungkyunkwan University, Suwon 16419, Republic of Korea; ^4^Department of Health Sciences and Technology, Samsung Advanced Institute for Health Sciences and Technology (SAIHST), Sungkyunkwan University, Seoul 06351, Republic of Korea; ^5^Breast Cancer Center, Samsung Medical Center, Seoul 06351, Republic of Korea; ^6^Department of Physiology, Ajou University School of Medicine, Suwon 16499, Republic of Korea; ^7^Biomedical Institute for Convergence at SKKU (BICS), Sungkyunkwan University, Suwon 16419, Republic of Korea

## Abstract

Chemoresistance is one of the major causes of therapeutic failure in breast cancer patients. In this study, the mechanism of chemoresistance in human triple-negative breast cancer (TNBC) cells (MDA-MB-231) induced by doxorubicin (DOX) gradient was investigated. These DOX-resistant cells showed higher drug efflux rate, increased anchorage-independent growth when cultured in suspension, and increased tumor-forming ability in nude mice, compared to the wild-type MDA-MB-231 cells. RNA sequencing analysis showed an increase in the expression of genes involved in membrane transport, antiapoptosis, and histone regulation. Kaplan-Meier plot analysis of TNBC patients who underwent preoperative chemotherapy showed that the relapse free survival (RFS) of patients with high HIST1H2BK (histone cluster 1 H2B family member k) expression was significantly lower than that of patients with low HIST1H2BK expression. Quantitative real-time PCR confirmed that the level of HIST1H2BK expression was increased in resistant cells. The cytotoxicity analysis showed that the DOX resistance of resistant cells was reduced by treatment with a histone deacetylase (HDAC) inhibitor. Our results suggest that, in DOX-resistant cells, HIST1H2BK expression can be rapidly induced by the high expression of genes involved in membrane transport, antiapoptosis, and histone regulation. In conclusion, chemoresistance in MDA-MB-231 cells can occur in a relatively short period by DOX gradient via this previously known mechanism of resistance, and DOX resistance is dependent on the specificity of resistant cells to HDAC.

## 1. Introduction

Breast cancer is largely classified into four subtypes depending on the molecular expression of cell surface receptors: normal-like, luminal (estrogen receptor-positive), epidermal growth factor receptor 2- (HER2-) enriched, and basal-like [1]. Triple-negative (estrogen receptor-negative, progesterone receptor-negative, and HER2-negative) breast cancer (TNBC) shares many of the properties of basal-like breast cancer. Based on these classifications, the therapeutic target and prognosis of each subtype differ [2]. TNBC has no known therapeutic targets to date, and as chemotherapy and radiotherapy are the standard clinical therapies for TNBC, tolerance to chemotherapy severely affects the prognosis of the patient.

Chemoresistance is a major cause of treatment failure in a variety of carcinomas and can be divided into primary resistance and acquired resistance. Primary resistance is a de novo lack of therapeutic response, whereas acquired resistance arises during the course of chemotherapy. The mechanisms of acquired resistance vary depending on the type of chemotherapeutic agent and patient, making it difficult to predict [3]. The following mechanisms promote direct or indirect resistance against anticancer drugs in human cancer cells: drug target alteration, drug inactivation, cell death inhibition, DNA damage repair, drug efflux, epigenetic alterations, and epithelial-mesenchymal transition (EMT) [4]. When using signaling blockers to inhibit cancer growth and survival, cancer cells can activate new pathways that bypass the signal. It is for this reason that many preclinical and clinical studies using single target antibodies and small molecule inhibitors have failed.

Doxorubicin (DOX) is widely used for chemotherapy as it kills rapidly proliferating cancer cells by targeting topoisomerase II [5]. The mechanisms of multidrug resistance signaling are known to be related to cell survival and growth and include the following signaling pathways: (i) G protein-coupled receptor (GPCR)/RAS/RAF/mitogen-activated protein kinase (MEK; also known as MAP2K, MAPKK)/ extracellular regulated kinases (ERK), (ii) IL6 (interleukin 6)/Janus kinase (JAK)/signal transducer and activator of transcription (STATs)/mechanistic target of rapamycin kinase (mTOR), and (iii) HER2/phosphoinositide 3-kinase (PI3K)/AKT. These signaling cascades stimulate the expression of transporter genes (ATP-binding cassette super-family G member 2 (ABCG2), survival genes (STAT3, HER2), transcription factors (AP-1 and NF-*κ*B, nuclear factor-kappa-B), and epigenetic genes (HDACs, histone deacetylases) [6]. Increased IL6 and IL8 secretion and STAT3 activity are important in the development of resistance [7–9]. Recent studies have reported that tumor dormancy can cause recurrence after a long period of time in patients who have had cancer and undergone chemotherapy [10, 11]. Tumor dormancy allows cancer cells to survive by acquiring new mutations, altering gene expression, slowing proliferation, and decreasing metabolism, thereby reducing the effects of anticancer drugs.

HDACs can regulate diverse cellular processes and gene expression through chromatin remodeling [12]. Dysregulated HDAC expression and genetic mutations are involved in stimulating neoplastic transformation in various types of cancer, including breast cancer [13]. HDAC inhibitors are synthetic compounds that suppress HDAC activity. Suberoylanilide hydroxamic acid (SAHA, also known as vorinostat and Zolinza) is a broad-spectrum HDAC inhibitor that suppresses all zinc-dependent HDACs at a low nanomolar concentration [13–15]. Crystallographic studies have indicated that SAHA inhibits HDAC activity by interacting with its catalytic site [16]. SAHA is the first HDAC inhibitor to be approved for cancer therapy by the US Food and Drug Administration (FDA) and has been used alongside capecitabine in phase 1 and phase 2 clinical trials against advanced breast cancer [17]. The clinical efficacy of HDAC inhibitors as a monotherapy was shown to be limited in hematologic tumors and poor in solid tumors. However, the combinational therapy of HDAC inhibitors and chemotherapeutic agents promotes apoptosis and has shown promising results in preclinical studies [13].

Microfluidic chips can be used to mimic an* in vivo* microenvironment that allows cells to be grown three-dimensionally [18]. This chip has the advantage of being able to grow a smaller number of cells than conventional culture dishes and only requires a small volume of the culture medium or drug. Additionally, the researcher can modify the structure of the chip to control the cell microenvironment by varying the concentration gradient, coculture, or surface material. Previously, we demonstrated that DOX resistance can be produced in a glioblastoma cell line (U-87 MG) within 7 days by using a microfluidic chip, which we call a Cancer Drug Resistance Accelerator (CDRA) chip. The CDRA chip contains 488 hexagonal microchambers (diameter 200 *μ*m, height 40 *μ*m) surrounded by two microchannels with overlapping gradients of DOX and nutrients [19].

The purpose of this study was to find a new method for predicting and inhibiting early resistance in TNBC, which has a poor prognosis in the presence of resistance. We investigated the early development of DOX-resistant TNBC by inducing rapidly emerging chemoresistance using the CDRA chip and identified the causative resistance gene using transcriptomic analysis.

## 2. Materials and Methods

### 2.1. Cell Culture

The MDA-MB-231 human TNBC cell line was purchased from the ATCC (American Type Culture Collection, USA) and cultured in RPMI-1640 medium (HyClone, USA) supplemented with 10% fetal bovine serum (FBS) (HyClone), 2 mM glutamine, 100 IU/mL penicillin, and 100 *μ*g/mL streptomycin (Gibco, USA).

### 2.2. Fabrication of the Microfluidic Chip

**Figure 1:**
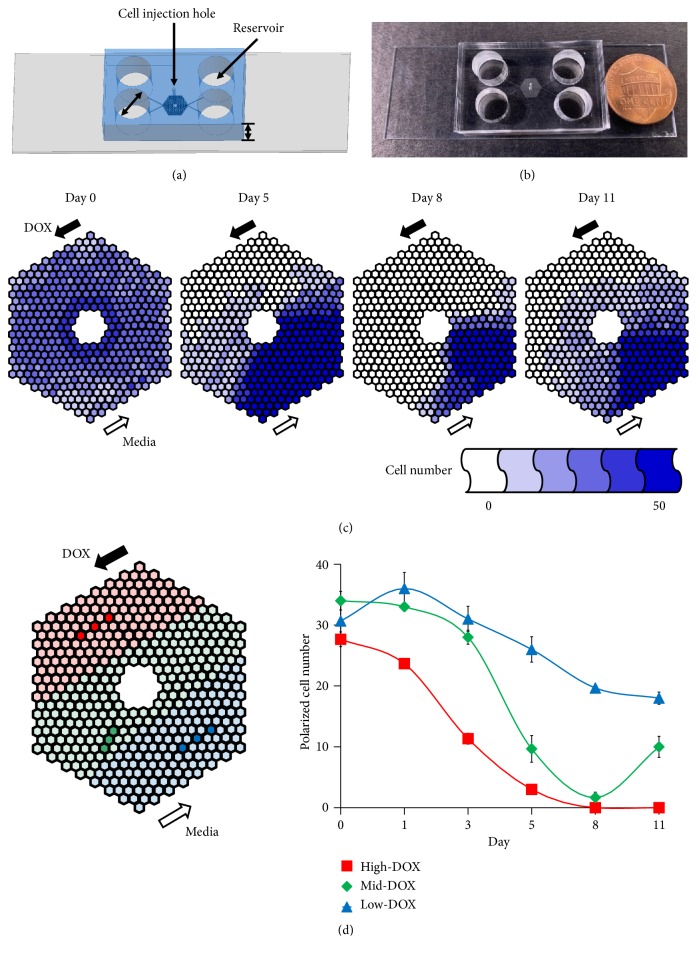
Tracking DOX concentration-gradient induced resistant cells on the chip. Schematic figure (a) and real image (b) of a CDRA chip. (c) Tracking the number of live cells in each chamber of a CDRA chip for 11 days. (d) Average number of polarized cells in the three different DOX concentration regions (red (high), green (mid), and blue (low)) over 11 days.

Soft lithography [19, 20] was used to make CDRA chips in polydimethylsiloxane (PDMS, Sylgard® 184 silicone elastomer; Dow Corning Co., USA). The PDMS prepolymer and curing agent mixture were combined at a ratio of 10:1 (w:w) and were cast onto Si mold containing the pattern of the CDRA chip and cured at 80°C for 2 h. A cell injection hole (700 *μ*m diameter) and four reservoirs (8 mm diameter) were punched into the chip (Figures 1(a) and 1(b)). The PDMS replica was treated with oxygen plasma to chemically bond it onto a glass slide (76 mm × 26 mm × 1 mm). The chip was autoclaved at 120°C for 1 h to prepare it for aseptic cell culture. The dimensions of the chip on glass slide were 32 mm (length) × 20 mm (width) × 9 mm (height). The chip contained a patterned array of 488 hexagonal microchambers, each with a diameter of 200 *μ*m and a height of 40 *μ*m. In the outermost chambers, 5-micron wide channels allowed media with or without DOX to flow into the interior microchambers. Each interior microchamber had three gates through which the cells could move into the connected chambers.

### 2.3. DOX Concentration Gradient in the Chip

To sterilize the chip, 70% ethanol (v/v) in sterile distilled water was injected through the cell injection hole at the center of the top of the chip using a needle-free syringe. The inside of the chip was then washed twice with sterilized PBS. To attach cells onto the bottom of the chip, 10 *μ*g/mL fibronectin (Sigma-Aldrich Co.) diluted in sterile PBS was injected via the injection hole and incubated at 37°C in a CO_2_ incubator for 1 h. Unbound fibronectin was washed away twice with PBS and then once with culture medium. To minimize fluid flow inside the chip prior to cell implantation, 200 *μ*L culture medium was added to each of the four reservoirs. A total of 5 × 10^4^ cells were suspended in 10 *μ*L culture medium and injected via the cell injection holes, which were then plugged using sterilized stainless pins to prevent liquid evaporation. The chip was incubated overnight at 37°C in a CO_2_ incubator. The DOX concentration gradient was produced in the chip by adding 250 *μ*L of culture medium containing 1.5 *μ*M DOX to each reservoir and 50 *μ*L to waste reservoirs.

### 2.4. Cell Viability

To remove nonresistant cells, the cells collected from the chip were allowed to grow in a medium containing DOX (IC_50_, half maximal inhibitory concentration) for one week. To assess the viability of wild-type (WT) and doxorubicin-resistant (DOXR) cells, about 1,000 cells were seeded in 96-well plates with or without DOX (Cat#D1515, Sigma-Aldrich) at the indicated concentrations. The EZ-Cytox cell viability kit (Daeil Lab Service, Korea) was used to detect viable cells. After incubating the cells for five days, 10 *μ*L EZ-Cytox solution was added to each well and then incubated at 37°C for 3 h. The absorbance was measured at 450 nm using a microplate reader (Molecular Devices, USA). The percentage viability was determined by normalizing against the value of the cells cultured without DOX. The control groups were grown in a medium with 0.01% dimethyl sulfoxide (DMSO, Sigma-Aldrich) to dissolve DOX compounds. Each condition was repeated in triplicate.

To verify the effect of suberoylanilide hydroxamic acid (SAHA) (Sigma-Aldrich) on DOX sensitivity, 1,000 WT or DOXR cells were seeded in each well of a 96 well-plate. The next day, cells were pretreated with or without SAHA for 1 h and then treated with DOX (Cat#44583, Sigma-Aldrich) at the indicated concentrations with or without 10 nM SAHA. After five days, cell viability was assessed using the EZ-Cytox kit.

### 2.5. Determination of Doubling Time

A total of 5 × 10^4^ WT or DOXR cells were seeded in 35 mm culture dishes (Falcon, USA) and maintained in growth medium. Both cell types were collected using trypsin and counted using a Luna automated cell counter (Logos Biosystems, Korea) after 24, 48, and 72 h.

### 2.6. Quantitative Real-Time PCR (qPCR)

Total RNA was extracted from the cells using an RNeasy Mini kit (Qiagen, Netherlands) according to the manufacturer's instructions. The total RNA was synthesized as cDNA using a cDNA synthesis kit (Fermentas, USA) for qPCR, according to the manufacturer's instructions. Gene expression was quantified by qPCR using an iQ SYBR Green Supermix (Bio-Rad, USA) with 100 ng of cDNA per reaction. The primer sequences used were as follows: *β*-actin: forward, 5′-AAT CGT GCG TGA CAT CAA A-3′, reverse, 5′-AAG GAA GGC TGG AAA AGA GC-3′; IL6: forward, 5′-GTA CAT CCT CGA CGG CAT CT-3′, reverse, 5′-GTG CCT CTT TGC TGC TTT CA-3′; CSF2: forward, 5′-CAC TGC TGC TGA GAT GAA TGA AA-3′, reverse, 5′-GTC TGT AGG CAG GTC GGC TC-3′; CXCR4: forward, 5′-GCA TGA CGG ACA AGT ACA GGC T-3′, reverse, 5′-AAA GTA CCA GTT TGC CAC GGC-3′; HIST1H2BK: forward, 5′-CAC CAG CGC TAA GTA AAC TTG CCA-3′, reverse, 5′-AGA GGC CAG CTT TAG CTT GTG GAA-3′; HIST1H1C: forward, 5′-ACA CCG AAG AAA GCG AAG AA-3′, reverse, 5′-GCT TGA CAA CCT TGG GCT TA-3′. An annealing temperature of 60°C was used for all primers. The primer pairs were synthesized by Bioneer Co. (Korea). qPCR was performed in a 96-well plate using a LightCycler Nano (Roche, Switzerland) instrument. For the data analysis, the raw threshold cycle (C_T_) value for each sample was normalized to the housekeeping gene, *β*-actin, to obtain a normalized ∆C_T_ which was then calibrated to control samples to obtain ∆∆C_T_ values.

### 2.7. Western Blot

Cell lysates were prepared to detect the protein expression of cleaved PARP1. Equal amounts of protein (25 *μ*g) were boiled with Laemmli sample buffer (Bio-Rad, USA) for 5 min and electrophoresed on 10% sodium dodecyl sulfate-polyacrylamide (SDS-PAGE) gels. Separated proteins were then transferred to Immobilon-P polyvinylidene difluoride (PVDF) membranes (Millipore, USA) and blocked with 5% skim milk (Millipore) in Tris-buffered saline (TBS) containing 0.01% Tween-20 (TBST) for 30 min. The membranes were washed three times for 30 min in TBST and incubated with PARP1 (1:1,000 dilution, v/v, Cat#9542, Cell Signaling Technology, USA) and *β*-actin (1:2,000 dilution, v/v, Cat#sc-47778, Santa Cruz Biotechnology, USA) antibody in TBST buffer at 4°C overnight. The membranes were washed once every 10 min for 1 h in TBST and then incubated with secondary anti-rabbit (1:2,000 dilution, v/v, Cat#ADI-SAB-300, Enzo Life Sciences, USA) and anti-mouse (1:2,000 dilution, v/v, Cat#ADI-SAB-100, Enzo Life Sciences) HRP-conjugated antibodies for 1 h in TBST at room temperature. Primary and secondary antibodies were diluted with TBST and washed once every 10 min for 1 h. For visualization of the blots, the membranes were developed using D-Plus ECL Pico solution (Dongin Biotech, Korea).

### 2.8. DOX Efflux

A total of 1 × 10^4^ WT or DOXR cells were seeded in 4-well Lab-Tek II chamber slides (Thermo Fisher Scientific, USA). Multidrug resistance was determined by assaying the ability of the cells to extrude fluorescent doxorubicin hydrochloride (*λ*_ex_ = 470 nm, *λ*_em_ = 585 nm) (Cat#44583, Sigma-Aldrich). Cells were incubated in a growth medium with 5 *μ*M DOX at 37°C for 3 h; then the medium was replaced with normal culture medium. After 24 h, the 585 nm emission of intracellular DOX was analyzed using a fluorescence microscope (Nikon, Japan). The fluorescence intensity of 11 randomly selected cells in each condition was analyzed for each condition in WT and DOXR cells using the ImageJ (USA) image processing and analysis software.

### 2.9. Anchorage Independent Growth

A total of 1 × 10^5^ WT or DOXR cells were cultured on ultra-low-attachment 6-well plates (Corning, USA) with DMEM/F12 (Gibco) medium containing 2 ng/mL epidermal growth factor (EGF) (PeproTech, USA), 2 ng/mL FGF (PeproTech), B-27 supplement (1×) (Gibco), and 1% antibiotics (Gibco). After 8 days, the number of WT and DOXR spheroids with a diameter over 100 *μ*m was counted.

### 2.10. Orthotopic Xenograft

A total of 5 × 10^6^ WT or DOXR cells were orthotopically engrafted into the second mammary fat pad of anesthetized 9-week-old female Balb/c nude mice (Orient Bio, Korea) with a high Matrigel matrix concentration (Becton Dickinson, USA). The tumors were observed three times a week for three weeks. Tumor size was measured using digital calipers. All animal protocols were approved by the Institutional Animal Care and Use Committee at the Samsung Medical Center of Korea.

### 2.11. RNA Sequencing

Total RNA was extracted from WT and DOXR cells using the RNeasy Mini kit (Qiagen). RNA quality was confirmed using Agilent's 2100 Bioanalyzer system (Agilent Technologies, USA). RNA sequencing was performed using the Illumina NextSeq 500 sequencing platform (Illumina, USA). Biological functions were determined using the ingenuity pathways analysis (IPA) web-based bioinformatics software (Qiagen). A two-fold change in DOXR gene expression was used as the cut-off value for genes with a significant change in expression compared to the WT cells.

### 2.12. Clinical Data Analysis

Kaplan-Meier (KM) curves were generated using the KM plot software with a database of public microarray datasets (http://kmplot.com/analysis). The HIST1H2BK gene expression results of 3,951 patients were collected, of which a cohort of 95 patients was selected with basal-like subtypes and having undergone neoadjuvant chemotherapy [21].

### 2.13. Statistical Analysis

Statistical significance was determined using a Student's* t*-test. The results are presented as the mean ± SDM. All quoted p values were two-tailed and differences were considered statistically significant at *∗p* < 0.05 and *∗∗p* < 0.01. Statistical analysis was performed using Microsoft Excel.

## 3. Results

### 3.1. Rapid Emergence of DOX-Resistant Cells in the CDRA Chip

The day after seeding cells in the CDRA chip comprising 488 chambers, there were approximately thirty cells per chamber. On the first day after treatment with a concentration gradient of DOX, the cells exposed to a high DOX concentration (high-DOX regions) and an intermediate concentration of DOX (mid-DOX regions) were beginning to die. By day 8, most of the cells in both regions were dead (Figure 1(c)). The dead cells were swept away by fluid flowing to the outlet reservoirs. After day 8, the number of cells increased in the mid-DOX region, indicating that some cancer cells exposed to a low DOX (low-DOX regions) migrated towards the mid-DOX regions and proliferated at a DOX concentration that the cells could tolerate. The number of cells in the three chambers and three different DOX concentration regions (high-DOX, mid-DOX, and low-DOX) was counted for 11 days. The number of cells in the high-DOX region near the DOX inlet continually decreased as a result of DOX-induced cell death, whereas the number of cells in the low-DOX region near the pure medium inlet decreased more gradually as a result of the high cell density (Figure 1(d)). The number of cells in the mid-DOX region decreased until day 8 and then increased again until day 11 (Figure 1(d), S1). This may be due to the cancer cells that had acquired resistance migrating to the nutrient-rich, high-DOX region from the nutrient-deficient region (low-DOX) where cells were densely packed.

### 3.2. Characterization of DOXR Cells

**Figure 2:**
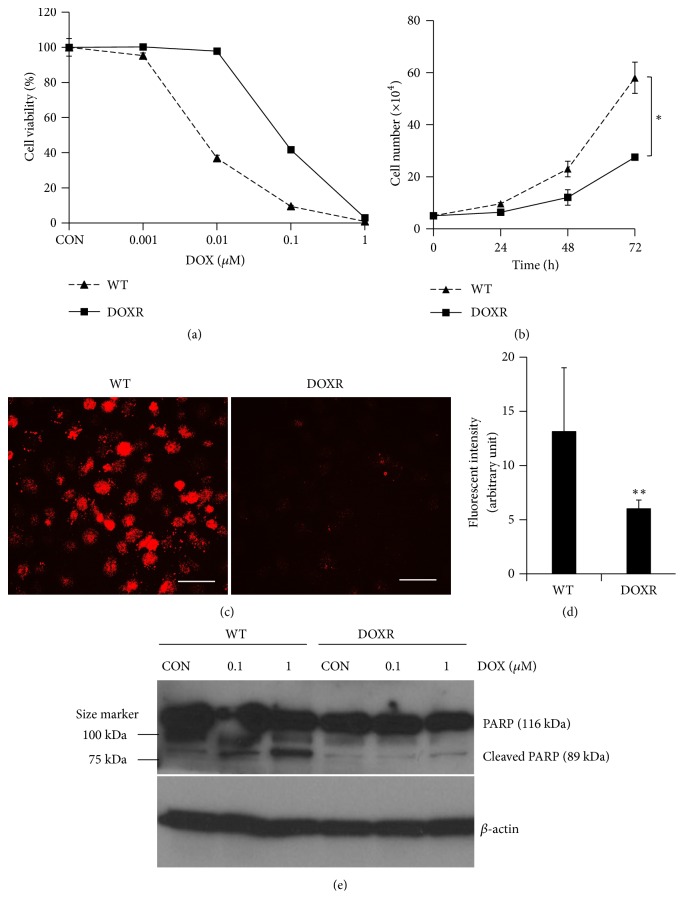
Characterization of DOXR cells. (a) DOX sensitivity of WT (IC_50_ = 8 nM) and DOXR (IC_50_ = 80 nM) cells. (b) Doubling time of WT and DOXR cells. *∗p* < 0.05, two-tailed Student's* t*-test. (c) DOX efflux ability of WT and DOXR cells. White bar = 50 *μ*m. (d) Red fluorescent intensity of WT and DOXR cells obtained by analyzing (c) using ImageJ software. *∗∗p* < 0.01, two-tailed Student's* t*-test. (e) PARP and cleaved PARP protein expression of DOX treated WT and DOXR cells. DOX treated for 48 h. Control (CON) groups were treated in growth medium with 0.01% DMSO (v/v) as vehicle. *β*-Actin was used as endogenous loading control.

To confirm the drug resistance of the cells in the chip, the cells were collected and their DOX sensitivity was measured. The cells were incubated in a medium containing DOX (IC_50_ of WT cells) for approximately 2 weeks. Under the same conditions, WT cells were found to be apoptotic and no longer proliferated (data not shown). The resistance of DOXR cells was approximately 10 times that of WT cells (Figure 2(a)), while their doubling time in a normal culture medium was half that of WT cells (Figure 2(b)). The decreased proliferation of DOXR cells has also been reported in a study using cell lines of other breast cancer subtypes [22]. Additionally, we compared the drug efflux rates between WT and DOXR cells as this is a major indicator of drug resistance. DOXR cells had significantly lower levels of intracellular DOX accumulation than WT cells (Figures 2(c) and 2(d)); therefore DOX-induced cytotoxicity had less of an effect on DOXR cells than on WT cells. To test the molecular impact of DOX on both cells, we identified cleaved PARP1 (poly (ADP-ribose) polymerase) protein expression as a cell apoptosis marker (Figure 2(e)). Cleaved PARP1 protein expression of WT cells increased by DOX treatment in a dose-dependent manner. In contrast, cleaved PARP1 protein expression did not change to DOX treatment in DOXR cells. This result supports the results of Figure 2(a) on DOXR cells, which better tolerate DOX-induced cytotoxicity.

### 3.3. Tumor Initiation Ability of DOXR Cells

**Figure 3:**
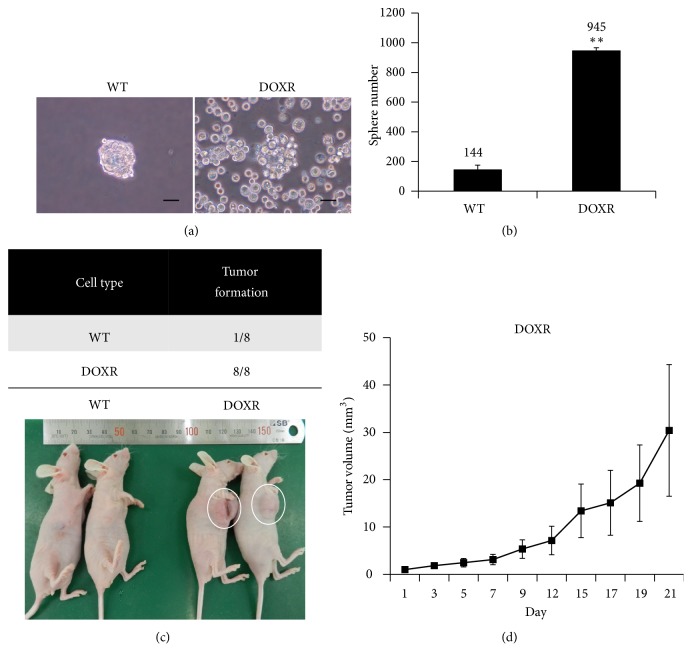
Tumor initiation ability of WT and DOXR cells. (a) Images of WT and DOXR cell sphere acquired at day 8. Black bar = 100 *μ*m. (b) Number of WT and DOXR cell spheres counts at day 8. *∗∗p* < 0.01, two-tailed Student's* t*-test. (c) Tumorigenicity of WT and DOXR cells in the orthotopic xenograft after 3 weeks. (d) Tumor volume of DOXR cell-injected xenograft (N = 8).

In order to test the anchorage-independent growth of DOXR cells, sphere formation and proliferative capacity were evaluated by suspending the cells in an ultra-low-attachment cell culture dish (Figure 3(a)). The sphere number of the DOXR cells was significantly higher than that of the WT cells (Figure 3(b)). Most single WT cells died over time and only some cells formed the spheroid, while single DOXR cells did not die and were eventually divided to form spheroids. In terms of morphology, DOXR cells were more irregularly shaped than WT cells. WT and DOXR cells were orthotopically transplanted into immune deficient mice to validate the enhanced anchorage-independent growth of DOXR cells observed* in vitro*. After 21 days, WT cells had only formed tumors in one of the eight mice, while DOXR cells had formed tumors in all of the mice (Figures 3(c) and 3(d)). These results are consistent with the* in vitro* results and demonstrate that resistant cells acquire not only DOX resistance but also increased tumorigenic capacity.

### 3.4. mRNA Expression Profiles of WT and DOXR Cells

**Figure 4:**
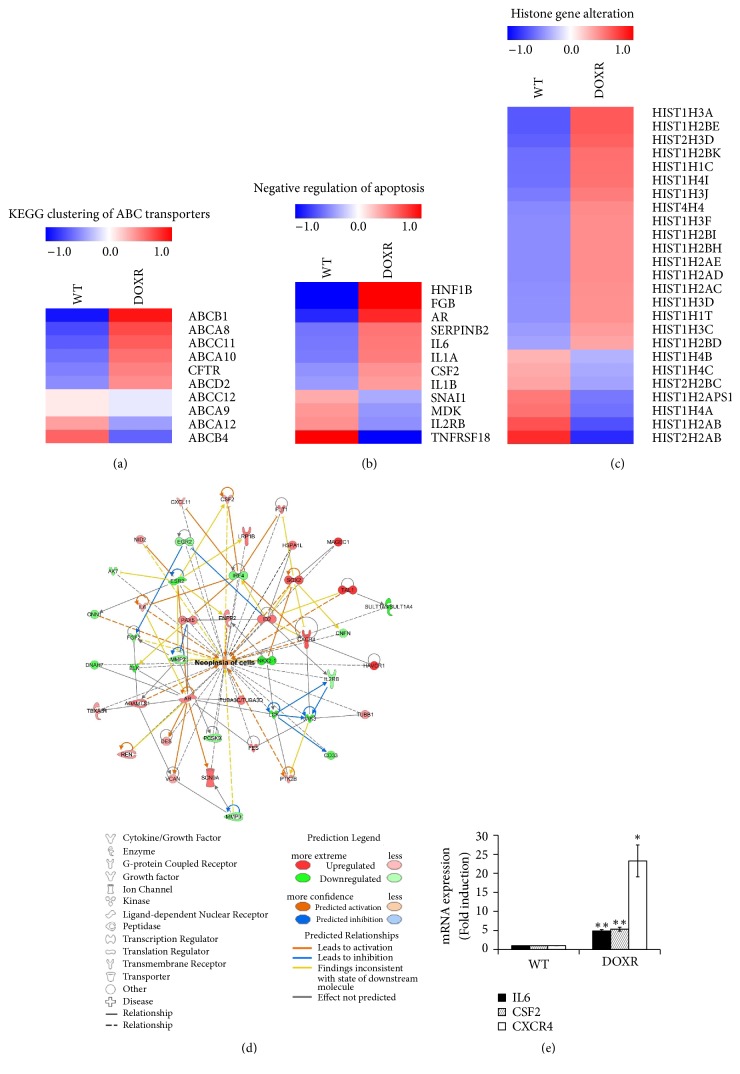
RNA sequencing analysis of WT and DOXR cells. (a) KEGG clustering of ABC transporters. (b) Negative regulation of apoptosis. (c) Histone gene alteration. Genes with more than a 1.5-fold-change were selected. (d) IPA analysis of WT and DOXR cells. The analysis employed a fold-change cut-off value of ± 2. Red and green areas indicate metabolite concentrations that were increased and decreased compared to the WT group, respectively. Orange and blue areas indicate prediction as activation and inhibition, respectively. (e) qPCR validation of neoplasia-related gene expression. *∗p* < 0.05, *∗∗p* < 0.01, two-tailed Student's* t*-test.

RNA sequencing analysis was performed to analyze the gene alteration of DOXR cells (Figures 4(a)–4(c)). IPA analysis, with a fold-change cut-off value of ± 2, revealed that the expression of cell survival, proliferation, antiapoptosis, and neoplasia-related genes was increased (Figure 4(d)). IPA also confirmed that IL6, CSF2, and CXCR4 were among the genes with increased expression as determined by qPCR (Figure 4(e)). The expression of ABC transporter genes, such as ABCB1 and AB CA8, and antiapoptosis-related genes was also increased in resistant cells. Interestingly, HIST1H2BK levels were found to be higher in histone modification-related genes and were shown to be 2.5 times higher by qPCR (Figure 6(a)). This gene has recently been associated with tumor dormancy [23], which is one of the major causes of chemoresistance.

### 3.5. Clinical Relevance of HIST1H2BK

**Figure 5:**
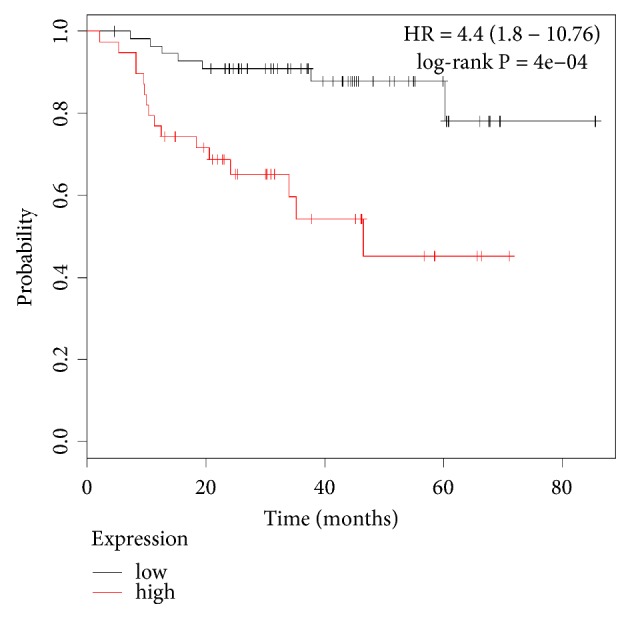
RFS of low (black line) and high (red line) HIST1H2BK expression in NAC.

To verify the clinical significance of the HIST1H2BK gene identified by mRNA sequencing analysis, a web-based public KM plotter database was used (www.kmplot.com). Of the 3955 breast cancer patients, a KM plot of HIST1H2BK expression in 114 patients with TNBC who underwent preoperative chemotherapy revealed that the relapse free survival (RFS) of patients with high HIST1H2BK expression was significantly lower than those with low HIST1H2BK expression (Figure 5).

Breast cancer patients were treated with basal-like TNBC. N = 95: low = 56 and high = 39.

### 3.6. HDAC Inhibitor Restores DOX Sensitivity in DOXR Cells

**Figure 6:**
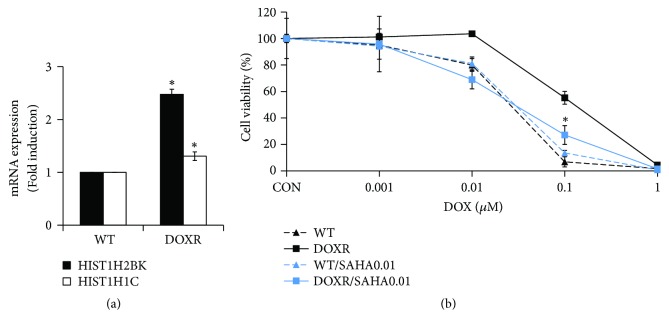
HDAC inhibitor (SAHA) restores DOX sensitivity in DOXR cells. (a) mRNA expression of HIST1H2BK and HIST1H1C in WT and DOXR cells. Significance compared with WT for each gene. (b) DOX sensitivity of WT and DOXR cells cultured with or without SAHA for 5 days. *∗p* < 0.05, two-tailed Student's* t*-test. Significance compared with DOXR and DOXR/SAHA0.01 at 0.1 *μ*M DOX. CON = control.

To examine the effects of histone gene overexpression in DOXR cells, we treated the cells with an HDAC inhibitor (SAHA) and DOX simultaneously. WT cells were not affected by DOX sensitivity when treated with SAHA; however, the DOX sensitivity of resistant cells was restored to the level of WT cells by SAHA treatment (Figure 6(b)). This shows that HDAC overexpression (Figure 6(a)) plays an important role in the early tolerance of cells to DOX.

## 4. Discussion

**Figure 7:**
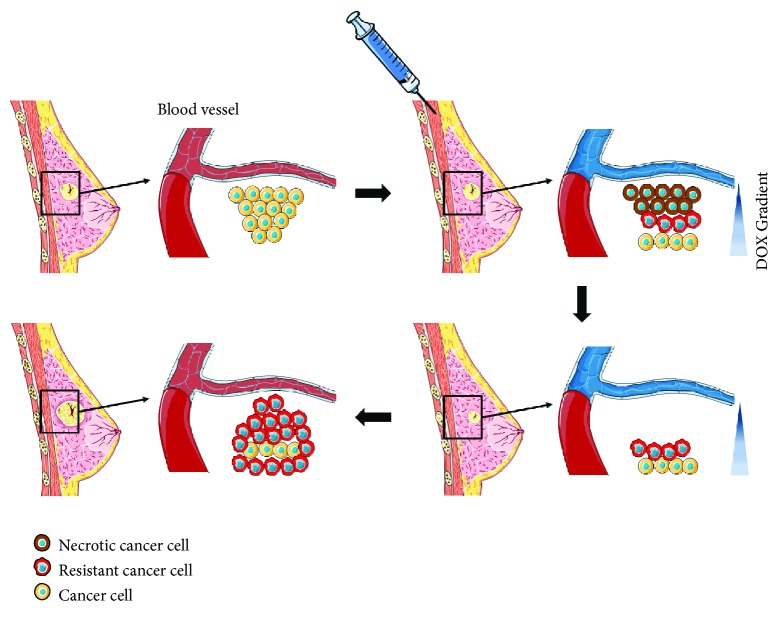
A schematic representation of the breast cancer patient's situation against chemotherapy corresponding to the acquisition of drug resistance derived from the CDRA concentration gradient chip. Art figure was made by recombining the images of the Creative Commons (creativecommons.org) released for free.

After the administration of the chemotherapy drug, a concentration gradient is generated around the blood vessels in a spatiotemporal manner. In solid tumors, the cells are densely packed and the permeability of the drug decreases toward the center of the tumor. Consequently, cancer cells existing in a region where they can withstand the drug concentration not only survive but also can adapt to the drug, as depicted in Figure 7. Many studies have reported on the relationship between acquired cancer resistance and long-term anticancer drug treatment [24, 25]. However, this study showed that such tolerance can be acquired over a relatively short period of time. Resistant cells showed decreased DOX sensitivity, decreased doubling time, and a high DOX-efflux rate, which are similar to previously reported DOXR cell characteristics. RNA sequencing analysis confirmed changes in gene expression, such as the increased expression of membrane transporter, antiapoptosis, and histone genes. HIST1H2BK gene was proven to significantly reduce the clinical prognosis of chemotherapy-treated TNBC patients. Concurrent treatment of an HDAC inhibitor with DOX restored the DOX sensitivity of resistant cells.

HDAC inhibitors have been tested in various cancers; however, HDAC inhibitor monotherapy appears to have reduced effects on cancer* in vitro* and* in vivo* [26]. It was reported that, for the combined treatment of an HDAC inhibitor and DOX, better anticancer effects could be obtained by blocking DNA repair and BCR-ABL-driven double strand break (DSB) repair mechanisms in animal models of leukemia [27].

In our previous study, we showed that STAT3 phosphorylation was induced by fibronectin (FN, an extracellular matrix glycoprotein) and CD44 (breast cancer stem cell marker which contributes to cell attachment, growth, migration, differentiation, and oncogenic transformation)* in vitro* using MDA-MB-468 and BT20 [28, 29]. Many other studies have reported that increased STAT3 phosphorylation in TNBC contributes to stemness, tumorigenesis, and cell survival [30–32]. Another study reported that the activation of LIFR-JAK1-STAT3 signaling in MDA-MB-231 cells limits their response to HDAC inhibitors [26]. Similarly, the HDAC inhibitor did not affect the DOX sensitivity of WT cells in this study; however, it recovered the DOX sensitivity of resistant cells. This suggests that the STAT3 expression pattern of resistant cells differs from that of WT cells, and that LIFR-JAK1-STAT3 signaling may be regulated by another mechanism. Future studies are required to elucidate more detailed mechanisms.

Recently, Kim* et al.* selected 49 signature genes of tumor dormancy from breast cancer cell line data and microarray results from patient samples and reported that the six most highly expressed genes were HIST1H2BK, STAT3, CTSD, SREBF1, IGFBP5, and DDR1 [23]. These results support the possibility that HIST1H2BK may be a tumor dormancy marker [23]. Dormant cells are more likely to survive chemotherapy as it targets rapidly proliferating cells, whereas dormant cells can grow for long periods of time and can reproduce when chemotherapy has finished.

Clinically, breast cancer chemotherapy can be classed as neoadjuvant chemotherapy (NAC) or adjuvant chemotherapy (AC) according to the time of treatment [33]. NAC is performed to reduce the size of the tumor before surgery if the tumor size of the patient is large. AC is performed to remove residual cancer cells after surgery. In this study, the tumor cells were treated with DOX in the CDRA chip to obtain resistant cells which were then subjected to genomic analysis, which is similar to the first round of NAC and genome analysis after sampling the tissue during surgery. Therefore, we used the data from NAC patients in the open web-based KM plot database. Tumors are diagnosed and chemotherapuetic drugs are administered, but cancer survives by various strategies such as decreased proliferation and increased drug efflux at tolerable dose. These resistant cells proliferate at a faster rate than in the environment where the drug is removed in the future, and the cancer may recur (Figure 7). The CDRA chip mimicked the appearance of resistant cells due to this concentration gradient and matched the clinical outcome for the HIST1H2BK gene. The next study will be to determine whether resistance is induced and correlated with clinical information using patient-derived cells.

## 5. Conclusions

This study demonstrated that chemoresistance can be acquired rapidly in MDA-MB-231 cells under a DOX concentration gradient in a microfluidic chip. In addition, we used genetic analysis to confirm that the histone gene, HIST1H2BK, is highly involved in early chemoresistance and showed that the HDAC inhibitor, SAHA, can restore DOX sensitivity.

## Data Availability

The data used to support the findings of this study are available from the corresponding author upon request.

## Supplementary Materials

Supplementary Materials Supplementary Figure S1: cell morphology on one of the marked chambers tracked for 11 days in Figure 1(d). Black bar = 100 *μ*m.
