# Supplementary material for: Chemoresistance in the Human Triple-Negative Breast Cancer Cell Line MDA-MB-231 Induced by Doxorubicin Gradient Is Associated with Epigenetic Alterations in Histone Deacetylase
Source: J Oncol. 2019 Jun 2;2019:1345026. doi: 10.1155/2019/1345026 (PMC6582875; doi:10.1155/2019/1345026)
Supplement: Supplementary Materials — Supplementary Figure S1: cell morphology on one of the marked chambers tracked for 11 days in Figure 1(d). Black bar = 100 μm. [file 1345026.f1.pdf]

Journal of Oncology

Chemoresistance in the human triple-negative breast cancer cell line MDA-MB-231 induced by doxorubicin gradient is associated with epigenetic alterations in histone deacetylase

Jeonghun Han,[1,2] Wanyoung Lim,[3] Daeun You,[4] Yisun Jeong,[4] Sangmin Kim,[5] Jeong Eon Lee,[4,5] Tae Hwan Shin,[6] Gwang Lee,[6] and Sungsu Park[2,7*]

[1]Regenerative Medicine and Cell Therapy Institute, Seoul National University Hospital Bundang, Seongnam 13620, Korea

[2]School of Mechanical Engineering, Sungkyunkwan University, Suwon 16419, Korea

[3]Department of Biomedical Engineering, Sungkyunkwan University, Suwon 16419, Korea

[4]Department of Health Sciences and Technology, Samsung Advanced Institute for Health Sciences and Technology (SAIHST), Sungkyunkwan University, Seoul 06351, Korea

[5]Breast Cancer Center, Samsung Medical Center, Seoul 06351, Korea

[6] Department of Physiology, Ajou University School of Medicine, Suwon 16499, Korea

[7]Biomedical Institute for Convergence at SKKU (BICS), Sungkyunkwan University, Suwon 16419, Korea

[*] Correspondence should be addressed to Sungsu Park; School of Mechanical Engineering, Sungkyunkwan University, Seobu-ro, Suwon 16419, Kyunggi-do, Republic of Korea/Email: nanopark@skku.edu/ Tel: +82-31-290-7431/Fax: +82-31-290-5889

Supplementary material:

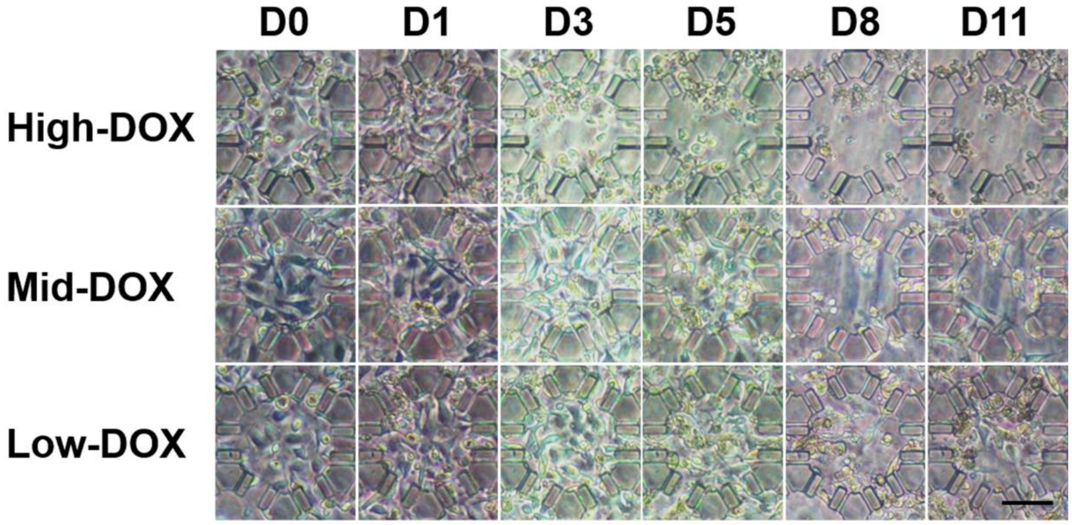

Figure S1: Cell morphology on one of the marked chambers tracked for 11 days in Figure 1(e). Black bar = 100 μm.
